# The Evolving Roles of Cardiac Macrophages in Homeostasis, Regeneration, and Repair

**DOI:** 10.3390/ijms22157923

**Published:** 2021-07-25

**Authors:** Santiago Alvarez-Argote, Caitlin C. O’Meara

**Affiliations:** 1 Department of Physiology, Medical College of Wisconsin, Milwaukee, WI 53226, USA; salvarez@mcw.edu; 2 Cardiovascular Center, Medical College of Wisconsin, Milwaukee, WI 53226, USA; 3 Genomics Sciences and Precision Medicine Center, Medical College of Wisconsin, Milwaukee, WI 53226, USA

**Keywords:** macrophages, cardiac homeostasis, myocardial infarction, inflammation, regeneration, monocytes

## Abstract

Macrophages were first described as phagocytic immune cells responsible for maintaining tissue homeostasis by the removal of pathogens that disturb normal function. Historically, macrophages have been viewed as terminally differentiated monocyte-derived cells that originated through hematopoiesis and infiltrated multiple tissues in the presence of inflammation or during turnover in normal homeostasis. However, improved cell detection and fate-mapping strategies have elucidated the various lineages of tissue-resident macrophages, which can derive from embryonic origins independent of hematopoiesis and monocyte infiltration. The role of resident macrophages in organs such as the skin, liver, and the lungs have been well characterized, revealing functions well beyond a pure phagocytic and immunological role. In the heart, recent research has begun to decipher the functional roles of various tissue-resident macrophage populations through fate mapping and genetic depletion studies. Several of these studies have elucidated the novel and unexpected roles of cardiac-resident macrophages in homeostasis, including maintaining mitochondrial function, facilitating cardiac conduction, coronary development, and lymphangiogenesis, among others. Additionally, following cardiac injury, cardiac-resident macrophages adopt diverse functions such as the clearance of necrotic and apoptotic cells and debris, a reduction in the inflammatory monocyte infiltration, promotion of angiogenesis, amelioration of inflammation, and hypertrophy in the remaining myocardium, overall limiting damage extension. The present review discusses the origin, development, characterization, and function of cardiac macrophages in homeostasis, cardiac regeneration, and after cardiac injury or stress.

## 1. Introduction

In the late 1880s Ilya Metchnikoff first described macrophages as phagocytic cells and key mediators in the phagocytosis theory [1]. Although cells engulfing foreign and deleterious particles and pathogens were observed before Metchnikoff, it was he who envisioned a network of phagocytes distributed through the organism. He described these cells to actively patrol and survey their microenvironments; encounter and distinguish innocuous from noxious material and/or pathogens; and actively phagocytose the latter, protecting the organism and maintaining homeostasis. In 1908 Metchnikoff received the Nobel Prize in Physiology or Medicine for his work. Decades later in the 1970s [2] this phagocytic theory constituted the basis for developing the mononuclear phagocytic concept, which classified promonocytes in the bone marrow (which are the first-identified cell of the mononuclear phagocytic system that is multiplicative and by dividing creates two monocytes), monocytes in the blood, and macrophages in tissues as part of the same developmental lineage that culminated in macrophages. Here, macrophages are considered a mononuclear highly phagocytic cell, different from polymorphonuclear phagocytes, and a key mediator of the innate immunity that mediates the phagocytosis of pathogens in multiple tissues [2].

For years macrophages were thought to develop exclusively from circulating monocytes that infiltrated tissues in the presence or absence of inflammation and became tissue-resident macrophages [3,4]. As we have learned more about the heterogeneity and lineages of macrophages, we now know that, in addition to monocyte-derived macrophages, many tissue-resident macrophage populations develop prior to the onset of hematopoiesis. These subpopulations of tissue-resident macrophages are broadly distributed throughout virtually all tissues during homeostasis and first appear during early embryogenesis [5,6,7,8]. Tissue-resident macrophages are defined as macrophages that perform homeostatic functions and reside in the tissue in the absence of injury or inflammation, and either arise independent of monocyte lineage and hematopoiesis, or are seeded from circulating monocytes. New research tools that look at macrophages at the transcriptomic or proteomic level, have unveiled the heterogeneity of these tissue-resident cells, describing how they exist as clusters of subpopulations with distinct and overlapping functions [9,10,11,12,13]. These cells perform constant surveillance and phagocytosis of foreign and deleterious cells and material that may alter the normal function of their microenvironment [8,14,15,16,17,18,19,20,21,22]. Examples of tissue-resident macrophages include Kupffer cells found in the liver and microglia located throughout the brain, as well as peritoneal, lung, splenic red pulp, and bone marrow (BM) macrophages [5,6,7,8,18,22]. Relatively recent research demonstrates the importance of embryonic and monocyte-derived cardiac-resident macrophages in cardiac homeostasis and after cardiac injury, beyond their phagocytic capacity. Here, we present a detailed review of the literature describing the ontogeny, development, characterization, and role of cardiac-resident macrophages in both homeostasis and after injury.

## 2. Origin and Development of Cardiac Resident Macrophages

**Figure 1:**
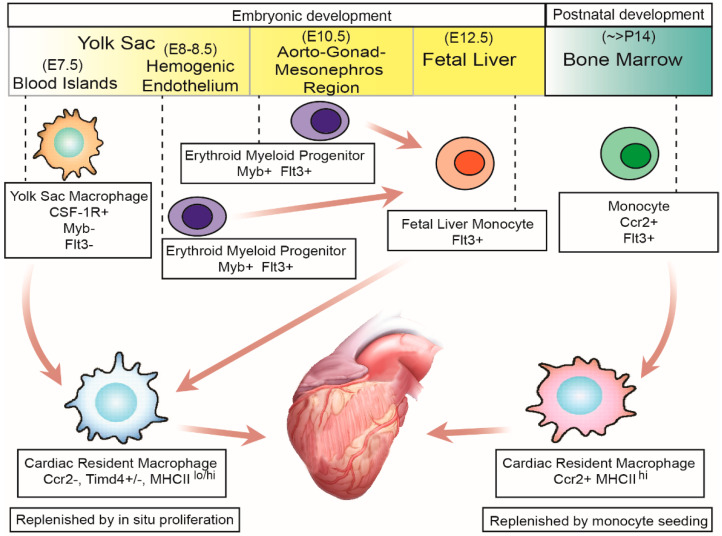
Derivation of cardiac-resident macrophages during embryonic and postnatal development. During embryonic development, cardiac-resident macrophages are derived from either yolk-sac macrophage-derived or fetal liver monocyte-derived cardiac-resident macrophages. The former develop, starting around embryonic day 7.0 (E7.0), and migrate to the heart at E7.5. The latter are derived from the erythroid myeloid progenitors from the hemogenic endothelium of the yolk sac (E8.0–8.5) and from the hemogenic endothelium of the aorto-gonad and mesonephros (AGM) region (E10.5). Fetal liver monocyte production starts at E12.5. Embryonic-derived cardiac-resident macrophages endure in the heart primarily by proliferation in situ. Bone marrow monocyte-derived cardiac-resident macrophages appear in the heart starting around postnatal day 14 (P14) and endure in the heart by continuous monocyte seeding from the circulation.

In the heart, the presence of multiple cardiac-resident macrophage subpopulations, with both specific and overlapping functions, has been identified [10,17,23,24,25,26]. This has been achieved using the following cell surface markers: leukocyte common antigen (CD45); integrin alpha M (CD11b), present in cells from myeloid origin and natural killer cells; and adhesion G protein-coupled receptor E1 (F4/80), a receptor broadly distributed in murine macrophages [27] of which two clusters can be identified: CD11b^lo^ F4/80^hi^ and CD11b^hi^ F4/80^lo^ macrophage clusters [17]. The CD11b^lo^ F4/80^hi^ cluster appears in the heart after primitive hematopoiesis around embryonic day 7.5 (E7.5) (see Figure 1). These macrophages are derived from yolk-sac macrophage precursors that develop in blood islands of the yolk-sac, a process dependent on the colony stimulating factor 1 (Csf-1) and its receptor (Csf-1R) but independent of myeloblastosis oncogene (c-Myb), which is a transcription factor required for definitive hematopoiesis [5,7,8,28,29,30,31,32]. The CD11b^hi^ F4/80^lo^ cluster appears after transient-definitive and definitive hematopoiesis (see Figure 1). Transient-definitive hematopoiesis starts around E8.5 in the hemogenic endothelia of the yolk-sac with the generation of erythroid-myeloid progenitors (EMP) that migrate to the fetal liver once circulation is established. EMP develop into fetal liver monocytes that subsequently migrate and colonize other tissues and differentiate into tissue-resident macrophages, including CD11b^hi^ F4/80^lo^ cardiac-resident macrophages. The last wave of embryonic hematopoiesis, or definitive hematopoiesis, begins at E10.5 in the hemogenic endothelia of the aorto-gonad mesonephros (AGM) region, where EMP develop and migrate to the liver and differentiate into fetal liver monocytes [4,5,7,8,17,18,33,34].

In addition to these markers, cardiac-resident macrophages can be subclassified according to their low or high expression of major histocompatibility complex II (MHC II^lo^ or MHC II^hi^) and expression of C-C motif chemokine receptor 2 (Ccr2), either positive or negative (Ccr2+ and Ccr2−) (see Table 1 and Figure 1). MHC II mediates antigen presentation to the T-Lymphocytes and the activation of adaptative immunity, while Ccr2 is the receptor of monocyte chemoattractant protein 1 (Mcp-1 or Ccl2), which is the main chemokine that mediates monocyte infiltration to multiple tissues during acute and persistent inflammation [35,36,37,38,39]. Classification using these markers is useful since Ccr2− macrophages are derived from yolk-sac precursors independent of monocytes, while most Ccr2+ macrophages develop from monocytes from the bone marrow or extramedullary tissues postnatally [10,17,23,26,40] (see Figure 1). Ccr2 expression is an important classification marker since recent research has demonstrated how Ccr2+ and Ccr2− macrophages have divergent functions in the healing heart after injury, discussed later [17,23,37,41]. Another cell surface marker useful to distinguish embryonic versus monocyte-derived cardiac-resident macrophages is T-cell immunoglobulin and the mucin domain containing 4 (Timd4) [10,42], as this receptor is highly expressed in embryonic-derived cardiac-resident macrophages but not in monocyte-derived macrophages [10].

Embryonic cardiac-resident macrophages survive in the heart throughout life by in situ proliferation, without contribution from circulating monocytes. One mechanism that has been shown to contribute to cardiac-resident macrophage proliferation is the activation of the scavenging receptor type A (Scaf1), which binds to multiple ligands including oxidized LDL, bacterial components from Gram negative and positive bacteria, and β-amyloid, among others, which leads to the intracellular activation of myelocytomatosis oncogene (c-Myc) [42]. Another mechanism that promotes cardiac-resident proliferation is the sensing of tensile forces present in the contracting myocardium, which results in the activation of mitogen-activated protein kinase (MAPK) which particularly signals Mek 1/2 [43], since macrophages increase DNA synthesis when tensile forces are applied in vitro [44,45,46]; the inhibition of Mek 1/2 in in vitro and in vivo after MI reduces cell cycle activity in cardiac macrophages. Interestingly, cardiac-resident macrophages are highly proliferative in newborns, but the proliferative rate rapidly decreases with age. Specifically, 10–40% of cardiac-resident macrophages are in the S-phase of the cell cycle in newborn mice, in contrast to 20 day-old and 30 week-old mice where only 5–20% and less than 1% of cardiac-resident macrophages are in the S-phase, respectively [26,47]. Nonetheless, cardiac-resident macrophages maintain a high proliferative potential through adulthood as observed after cardiac stress and macrophage depletion experiments [17].

Fate-mapping experiments show that some Ccr2+ cardiac macrophages appear in the heart around embryonic age E14.5, and localize within the trabecular projections [48]. However, flow cytometry and histological experiments have shown that a more robust quantity of Ccr2+ macrophages appear in the heart starting at postnatal day 14 (P14) in mice [40,48]. These Ccr2+ macrophages are derived from monocytes which originate in the bone marrow or extramedullary tissues and show 50% replenishment by circulating monocytes within 3 weeks [10], depending on the Ccl2-Ccr2 signaling to infiltrate the heart [10,41,42]. Additionally, there is an increase in the Ccr2+ macrophage contribution to the total cardiac-resident macrophage pool with ageing [47]. A progressive increase in Ccr2+ cardiac-resident macrophages may contribute to the development and/or progression of cardiac-pathological conditions that also have a positive correlation with ageing, such as heart failure and heart failure exacerbation [49]; however, further research, which examines this possible correlation and pathophysiological mechanisms, is needed.

There are discrepancies regarding the contribution of embryonic-derived versus monocyte-derived macrophages to the total cardiac-resident macrophage pool. Molawi et al. studied the embryonic contribution to cardiac-resident macrophages using yellow-fluorescent protein (YFP) driven by tamoxifen-inducible Cre recombinase under the control of a fractalkine receptor (Cx3cr1^CreER^ R26-YFP) [47]. In more than 90% of cardiac-resident macrophages, Cx3cr1 is expressed [10,25,42,50,51]. By performing pulse-chase experiments, they found that in newborn mice ~35% of the total cardiac-resident macrophages were embryonic-derived, but this value decreased to ~18% in 5–6 week-old mice [47]. Additionally, when YFP was induced by tamoxifen administration in adult mice, 60% of macrophages persisted YFP+ 1 week after tamoxifen discontinuation but only 20% persisted YFP+ after 4 weeks of tamoxifen discontinuation. Meanwhile, 100% of brain microglia persisted YFP+ after 4 weeks. Collectively, these data indicate that only 20% of cardiac macrophages endure by in situ proliferation over the 4-week time period, while the remaining 80% are replenished by circulating monocytes. Conversely, Epelman et al. found that embryonic contribution to cardiac-resident macrophages highly depends on the subpopulation studied. When cardiac-resident macrophages from 20-week old mice were subclassified by the expression of Ccr2 and MHC II, ~65% of Ccr2− MHC II^lo^, ~83% of Ccr2− MHC II^hi^, and ~9% of Ccr2+ MHC II^hi^ were found to be embryonically derived [17], suggesting that cardiac-resident macrophages were very heterogenous in their origin and probably had distinct functions in the heart during homeostasis. More recent experiments performed by Dick et al. corroborate the results previously described. Using the pulse-chase approach with different mouse reporter lines these investigators found that after labeling cardiac-resident macrophages in 3-week-old mice and assessing them 20 weeks after tamoxifen discontinuation, on average ~78% of cardiac-resident macrophages were maintained by proliferation in situ [10,17]; however, when cardiac-resident macrophages were subclassified by Ccr2, MHC II, and Timd4 expression, ~90% of Ccr2− MHCII^lo^ Timd4+; ~75% of Ccr2− MHCII^hi^ Timd4−; and ~15% of Ccr2+ MHCII^hi^ Timd4− cardiac-resident macrophages endured by proliferation in situ [10] (see Table 1). These studies demonstrate the difference in origin of different subpopulations of cardiac-resident macrophages and the utility of Ccr2 and Timd4 to distinguish cardiac-resident macrophages that endure by proliferation in situ versus those that are maintained by continuous monocyte infiltration. 

## 3. Advancements in Cardiac Resident Macrophage Characterization

Tissue-resident macrophages are part of the mononuclear phagocytic cells that exist in multiple tissues throughout the body [22]. In the heart, cardiac-resident macrophages have been described using multiple cell-tracing models. The most widely used and validated model is the transgenic line Cx3cr1-green fluorescent protein (Cx3cr1^GFP/+^) whereby one allele of the gene Cx3cr1 is replaced with GFP, therefore all cells constitutively expressing Cx3cr1 are GFP positive (GFP+). This model shows that over 90% of cardiac-resident macrophages are GFP+ [10,25,26,50,51], making this a useful reporter model for identifying macrophage versus non-macrophage cell types in the heart. Leukocytes represent ~10% of the total non-cardiomyocyte cells in the heart [50]. Among the total leukocytes, macrophages comprised 65–80% of total leukocytes during homeostasis [25,26,43]. Cardiac-resident macrophages display a spindle-like shape with long cytoplasmic projections that are in contact with surrounding cardiomyocytes and other stromal cells. Using clarified cardiac tissue, it has been revealed that there are ~3 × 10^5^ cardiac-resident macrophages in the murine heart during homeostasis [51]. Notably, there is an even higher concentration of macrophages in the atrio-ventricular (AV) node and cardiac-central fibrous body [52]. Each cardiomyocyte is in contact with on average 4–5 cardiac-resident macrophages and each cardiac-resident macrophage can be in contact with up to five cardiomyocytes. Additionally, it is estimated that each cardiac-resident macrophage is in contact with at least one capillary [25]. All cardiac-resident macrophages are positive for the markers CD45, CD11b, F4/80, high-affinity Fc receptor of IgG (CD64), lysosomal associated membrane glycoprotein CD68, and scavenging receptor MER proto-oncogene tyrosine kinase (Mertk) [10,17,25,41,42]. Compared to microglia and spleen monocytes/macrophages, cardiac-resident macrophages have enriched expression of signature genes like the folate receptor 2 (Folr2); the lymphatic vessel endothelial hyaluronan receptor 1 (Lyve1); the scavenging receptor for the hemoglobin–haptoglobin complex (CD163); the insulin-like growth factor 1 (Igf1); the mannose receptor 1 (Mrc1 or CD206); resistin-like alpha (Retnla); and the LPS co-receptor CD14, which resemble an alternative activation phenotype that is associated with anti-inflammatory properties and wound resolution [53]. However, some genes have unique functions which are important in the context of cardiac homeostasis and development, such as Lyve1, implicated in adipose tissue angiogenesis [54] and lymphangiogenesis in the heart [55,56]; or Igf1, involved in coronary development and maturation during cardiac development [48]. Furthermore, the high expression of the scavenging receptors Mertk, CD206, CD163, and CD14 suggest a highly phagocytic capacity, which is fundamental to maintain a clean extracellular environment for proper myocardial performance [51].

Using the expressions of Ccr2, Timd4, and MHC II, cardiac-resident macrophages can be functionality-subclassified, at least to some extent (see Table 1 and Figure 1). The Ccr2− Timd4− MHC II^hi^ macrophages have also been shown to be important for antigen presentation and the activation of T-lymphocytes [17]. The Ccr2− Timd4+ MHC II^lo^ macrophages are responsible for the continuous surveillance of the myocardium, phagocytosis of apoptotic cardiomyocytes, and material derived from CMs [17,51,57]. The Ccr2+ Timd4− MHC II^hi^ macrophages are necessary for lymphocyte antigen 6 high (Ly6c^hi^) Ccr2+ Cx3cr1^lo^ monocytes to infiltrate the heart after cardiac injury [17,37,41,42]. The Ccr2+ Timd4− MHC II^hi^ macrophages also have increased inflammasome activity and IL1b expression compared to Ccr2− subpopulations [40], which adversely contributes to the development of ischemic heart failure [23,37,51]. Thus, Ccr2+ cardiac-resident macrophages display more pro-inflammatory phenotypes compared to the other subclassifications.

In addition to classifying cardiac-resident macrophages purely by cell surface markers, single-cell transcriptomics has more recently been employed. Using the single-cell RNA sequencing (scRNAseq) of cardiac macrophages isolated from uninjured mouse hearts, Dick et al. [10] classified cardiac-resident macrophages under homeostasis into 4 different clusters (see Table 2). A first cluster (scRNAseq 1) is defined by the high expression of the genes Timd4, Folr2, Lyve1, CD163, and Igf1; a second cluster (scRNAseq 2) is defined by the high expression of MHC II, F4/80, Cx3cr1, and CD14; a third cluster (scRNAseq 3) is defined by the high expression of Ccr2 and CD64; and a fourth group (scRNAseq 4) is defined by the high expression of interferon responsive proteins, such as the interferon-induced protein with tetratricopeptide repeats 1 and 3 (Ifit1 and Ifit3); the ubiquitin-like modifier Isg15; and the interferon regulatory factor 7 (Irf7). Ingenuity pathway and pseudo-temporal analysis revealed that scRNAseq 3 and 4 clusters have upregulated classical inflammatory pathways and are closely related to each other and to circulating monocytes. Meanwhile, the scRNAseq 2 cluster has increased translational-ribosomal pathways and antigen presentation functions, and the scRNAseq 1 cluster has upregulated angiogenesis, endocytosis, and lysosomal activity, essential functions in normal heart homeostasis and development.

In humans, cardiac macrophages have been characterized as a population that is broadly CD14, CD163, CD64, and CD68 positive. Additionally, using Human leukocyte antigen-DR isotype (HLA-DR) and CCR2 cell surface markers, two populations of macrophages and one population of monocytes can be identified: CCR2+ HLA-DR^hi^ and CCR2− HLA-DR^hi^, which are macrophages; and CCR2+ HLA-DR^lo^, which are monocytes [10]. HLA-DR^hi^ CCR2− macrophages have increased the expression of genes TIMD4, LYVE1, CD163, FOLR2, and IGF; while HLA-DR^hi^ CCR2+ macrophages and HLA-DR^lo^ CCR2+ monocytes have increased CCR2 and interferon-response element’s expression [10], which resembles cardiac-resident macrophage subpopulations and functions as described in mice. Similar to mice, studies done in samples from human patients suggest that CCR2 expression can also distinguish embryonic from monocyte-derived cardiac-resident macrophages [23].

## 4. Homeostatic Impact of Cardiac Macrophages

Extensive research has described macrophages as key mediators of the innate immune system and has established their role in the phagocytosis of endogenous and foreign threats to maintain homeostasis [16,20,21]. However, macrophages can be identified in early embryogenesis in multiple tissues (such as the brain, liver, skin, and the heart) [5,7,17,18,35,42] before hematopoiesis starts in the bone marrow, suggesting that their functions are beyond the strictly immunological. For example, stromal macrophages in the fetal liver and the bone marrow before and after birth, are known to contribute to the maturation of nucleated erythroblasts and immature myeloid-derived leukocytes, respectively [57] (reviewed in [58]). Intestinal macrophages, which are located in the smooth muscle layer and in proximity to the myenteric plexus, facilitate peristalsis [59], and Kupffer cells in the liver and splenic macrophages phagocytize senescent and dying erythrocytes to recycle iron, also providing a source of lipid ligands and iron for new erythrocyte formation (reviewed in [60]). Microglia in the brain have numerous homeostatic functions including mediating dopaminergic circuit maturation in the developing forebrain [61]; synapsis formation and pruning throughout the brain (reviewed in [62]); and contributing to the laminar localization of subsets of interneurons [61]. Within the heart, cardiac tissue-resident macrophages are becoming an intense area of interest as new research demonstrates their importance, not only during development and in homeostasis, but also after cardiac injury. In this section we discuss specific functions that have been attributed to cardiac-resident macrophages during development and in homeostasis.

### 4.1. Capillary Development and Lymphatic Network Maturation

Ccr2− cardiac-resident macrophages are located within the myocardium, near the epicardium and in close relation with newly forming capillaries, veins, and lymphatic vessels at early stages of embryogenesis (E14.5) [48,55]. The mouse genetic line Csf1^op/op^, which lacks tissue-resident macrophages derived from yolk sac progenitors, has an aberrant pattern of vasculature within the heart, characterized by an increased number of small diameter capillaries (<12 μm^2^), but fewer large-diameter capillaries (>12 μm^2^). Moreover, many of these smaller-diameter capillaries are not readily perfused in Csf1^op/op^ mice [48]. Additionally, adult Csf1^op/op^ mice have excessive branching from mid-size coronary vessels [48]. Unlike Ccr2− cardiac-resident macrophages, Ccr2+ cardiac-resident macrophages are not necessary for appropriate vasculature development. The Ccr2− macrophages were shown to synthesize and secrete Igf1, which induces capillary tube formation and endothelial cell migration in in vitro angiogenic assays [48], suggesting that Igf1 secretion by embryonic-derived cardiac-resident macrophages during embryogenesis is a mechanism that contributes to normal coronary development.

In addition to coronary development, cardiac-resident macrophages are essential for proper lymphangiogenesis and lymphatic vessel maturation in the heart. Cardiac-resident macrophages derived from yolk-sac precursors colocalize with lymphatic endothelial cells (LEC) [55,56] and function as chaperone cells, guiding the LEC to another nearby LEC in order to form lymphatic junctions, extending the lymphatic network of the heart [56]. Macrophage depletion during early embryogenesis prevents cardiac-resident macrophage seeding resulting in impaired lymphangiogenesis [56]. Importantly, it is proposed that cardiac-resident macrophages secrete hyaluronan, which promotes bridging among LEC in in vitro assays. Inhibition of hyaluronan by hyaluronidase impedes sprouting and bridging between LECs [56]. Interestingly, only cardiac-resident macrophages expressing Lyve1 contribute to lymphangiogenesis [55], which are cardiac-resident macrophages developed during primitive hematopoiesis in the yolk-sac [10,17,55,56]. Modulation of lymphangiogenesis by cardiac-resident macrophages is important not only from a developmental perspective, but also in the context of ischemic cardiac remodeling since lymphangiogenesis is a mechanism that regulates adverse remodeling and facilitates circulation of infiltrating immune cells and clearance of dead material from the ischemic heart after injury [63,64,65].

### 4.2. Electrical Conduction in the Heart

Within the atrial-ventricular (AV) node cardiac-resident macrophages are present at a relatively high abundance, which leads to the hypothesis that cardiac macrophages may play a role in electric conduction in the heart [52]. Additionally, some cardiac-resident macrophages are in direct contact with cardiomyocytes (CMs) through gap junctions, and synchronously depolarize with surrounding CMs, further supporting the idea of an active role in cardiac conduction.

Mathematical modeling suggests that cardiac-resident macrophages in contact with CMs through gap junctions decrease cardiac refractory time, resulting in overall enhancement of cardiac conductivity. A proposed mechanism is that cardiac-resident macrophages can work as bridges between CMs that are not in direct contact. Subtle depolarization of cardiac-resident macrophages’ resting membrane potential enhances cardiac conductivity in ex vivo experiments. On the other hand, depletion of connexin43 (Cx43) in macrophages, which is the main component of gap junctions between cardiac macrophages and CMs, resulted in an impairment of the AV node conduction [52].

In the case of macrophage depletion by diphtheria toxin receptor expression, driven by CD11b (CD11b-DTR) and the diphtheria toxin (DT), administration led to an initial robust cardiac-resident macrophage depletion, but a subsequent repopulation by circulating monocytes [26,52]. Despite the monocyte repopulation of cardiac macrophages, mice depleted of macrophages developed spontaneous first, second, and third degree AV blocks [52].

The development of AV blocks is accelerated by pressure overload, as demonstrated by experiments where cardiac macrophage is depleted using clodronate liposomes, or when the mouse line CX3CR1-DTR and DT administration before pulmonary artery banding resulted in sudden death a few hours after banding, due to the development of a complete AV block and ventricular arrest [66], which is not observed after the depletion of other immune cells including lymphocytes and granulocytes [66]. Amphiregulin (Areg) is a membrane protein that could be secreted by multiple cell types, including cardiac-resident macrophages [66]. In the heart, it was demonstrated that Areg, secreted by cardiac-resident macrophages and acting through the epidermal growth factor receptor (Egfr), promotes the phosphorylation of Cx43 in CMs, resulting in the correct localization of Cx43 in gap junctions between CMs and the enhancement of membrane permeability and cardiac conductivity [66].

These experiments exposed a highly specialized function of cardiac-resident macrophages as enhancers of cardiac conductivity. Moreover, it opens the possibility of targeting cardiac-resident macrophages as a therapeutic tool in arrythmia and AV blocks. Although this is a function of cardiac-resident macrophages and not monocyte-derived macrophages, further research may unveil how cardiac-resident macrophages mechanistically mediate this enhancement and if such mechanisms can be targeted in monocyte-derived macrophages to promote cardiac conductivity.

### 4.3. Mitochondrial Function

It is known that cardiac macrophages are widely distributed within the myocardium and are in close contact with CMs, and these cardiac macrophages show a high expression of scavenging receptor such as Mertk, CD206, CD14, and CD64 [25,42,51,57]. The expression of these receptors supports the idea that cardiac macrophages survey the myocardium and actively phagocytose waste material present in the extracellular compartment. CMs dispose of dysfunctional mitochondria and other organelles through excretion of small vacuole, termed “exopheres” [51]. The generation and secretion of these exopheres by CMs is promoted by CM autophagy. Interestingly, macrophages actively engulf and phagocytose these exopheres, maintaining a clean extracellular microenvironment, and this relationship is essential for the normal functioning of the heart [51]. Both macrophage depletion and the global depletion of Mertk, a phagocytic receptor, leads to an increased number of exopheres in the cardiac extracellular milieu, impairs cardiac–aerobic metabolism, and deteriorates cardiac systolic and diastolic function [51]. Additionally, cardiac exophere clearance by cardiac macrophages is an important mechanism ameliorating damage induced by cardiac stress [51]. This is demonstrated by experiments where CM autophagy and the production of exopheres is increased by cardiac stress, and this effect is exacerbated by macrophage depletion, which also results in more damage [51]. Targeting cardiac-resident macrophage phagocytic capacity after cardiac injury or stress is therefore a promising field of research for the maintenance of cardiac health.

## 5. Role of Cardiac Macrophages Following Adult Cardiac Injury

### 5.1. Ischemic Heart Failure

Heart failure is one of the most prevalent and important diseases in both the developed and developing world [49]. It is projected to affect over 8.5 million adults in the United States by 2030. The main contributor to this chronic disease is myocardial infarction (MI), which is characterized by irreversible ischemia to the myocardium and loss of functional CMs, which are replaced by a non-functional scar. MIs are highly prevalent; approximately 805,000 adults had an MI in 2020 in the United States [49]. While many survive the initial event, there is a continuum by which the heart repairs following injury. It is well known that immune response plays a significant role in the post-cardiac injury response [24,67,68,69,70,71], and this immune cell response is highly dynamic and characterized by an initial inflammatory phase, where pro-inflammatory neutrophils and monocytes infiltrate the myocardium and injured region [67,69,72,73,74,75,76,77]. Later there is a profibrotic and anti-inflammatory response, mainly orchestrated by infiltration and the expansion of anti-inflammatory and reparative macrophages [67,69,72,73,74,75,76,77]. Distinguishing the molecules and mechanisms that govern this dynamic response is, not surprisingly, difficult; however, much effort is currently underway, since the manipulation of many components of this response have seen promising results in promoting cardiac healing [42,70,76,78,79,80,81,82].

After MI, there is early infiltration of neutrophils which peaks 1–2 days post-injury [72,73,74]. Posteriorly, a mononuclear phagocytic response takes place, characterized by an initial infiltration of Ccr2+ Cx3xr1^lo^ Ly6c^hi^ monocytes into the heart, primarily to the infarcted and peri-infarcted zones [26,69,75,76], that differentiate into inflammatory macrophages. As a result, the proportion of cardiac-resident macrophages that are maintained by proliferation in situ within the total pool of macrophages present in the heart after injury, is reduced from ~78% to ~4% [10]. Monocytes that infiltrate the heart originate from the bone marrow or from a monocyte pool that exists in the spleen [69,83,84]. Nonetheless, immunofluorescence and histological analyses revealed that after MI cardiac-resident macrophages were depleted only in the infarcted zone, whereas there was an increase in the peri-infarcted zone and no change in the remote zone [10]. These analyses are consistent with findings that cardiac stress induced by angiotensin II administration resulted in general cardiac-resident macrophage proliferation [17]. After 4 weeks, cardiac-resident macrophage abundance is the same in infarcted, peri-infarcted, and remote zones, and the same as in uninjured hearts [10]. However, there is an increased overall abundance of total macrophages in hearts 4 weeks after injury, and despite a robust recovery of cardiac-resident macrophages that are maintained by proliferation in situ, these represent only 48% of the total macrophage pool within the heart, compared to ~78% in the uninjured heart [10]. Thus, it is clear that some monocytes infiltrate the heart after injury, differentiate into macrophages, and persist in the heart for a long period [10,17,26]. However, it is unclear if these macrophages adopt the same phenotype and functions of cardiac-resident macrophages present before injury, or if they contribute to ischemic heart failure development and progression [85]. There is also a lack of studies that track cardiac-resident macrophage abundance and impact beyond 1 month after MI. This is critical since current evidence suggests that the cardiac-resident macrophage pool is permanently modified by a significant increase in monocyte-derived macrophages, which chronically persist in the heart after MI and may contribute differently to myocardium remodeling at the chronic stage. Importantly, it has been demonstrated that, at the chronic stage of ischemic heart failure (12 weeks after MI), adaptative immunity plays a role in myocardium remodeling and function impairment, which is characterized by the presence of effector T-cells in the heart and mediastinal lymph nodes and high affinity IgG anti-heart antibodies deposition in the myocardium [86]. It is unknown if there is an interaction between cardiac-resident or monocyte-derived macrophages and these antibodies and/or effector T-cells, and if these relationships contribute to ischemic heart failure progression. Nonetheless, it is known that there is a negative correlation between CCR2+ cardiac-resident macrophage abundance and response to therapy in patients with chronic ischemic heart failure that were treated with a left-ventricular assisting device (LVAD) [23]. However, this study didn’t distinguish between CCR2+ cardiac-resident macrophages normally present in homeostasis versus monocyte-derived macrophages that infiltrated after the ischemic event.

Single-cell transcriptomic analysis 11 days after MI in mice, demonstrated that the majority of macrophages in the heart after myocardial infarction are monocyte-derived; however, some of these monocyte-derived macrophages adopt the transcriptional profile of the cardiac-resident macrophages present in homeostasis, but there are some transcriptional profiles that remain unique to cardiac-resident macrophages [10]. The authors described 11 transcriptionally distinct macrophage clusters after MI [10], four of which are present in homeostasis and were previously discussed. Macrophage clusters that appear after MI are enriched in genes such as membrane-spanning 4-domains subfamily A member 7 (Ms4a7); secreted phosphoprotein 1 (Spp1); kinesin family member 2A (Kif2;); platelet factor 4 (Pf4); coagulation factor XIII, A1 subunit (F13a1); platelet glycoprotein 4 (CD36); and Ccr2, among others. These genes are commonly enriched in monocytes or are related to coagulation; tissue injury and remodeling; leukocyte migration; wound-healing regulation; Tnf production; and phagocytosis, which are highly prevalent scenarios in the healing myocardium. Interestingly, genes like Timd4, Folr2, Lyve1, Retnla, and CD163 are enriched only in the macrophage clusters present in homeostasis. Thus, monocyte-derived macrophages that infiltrate the heart after injury and remain there chronically adopt some of the transcriptional profiles displayed by cardiac-resident macrophages. However, some transcription patterns remain unique to cardiac-resident macrophages present in homeostasis and suggest that monocyte-derived macrophages are more fibrotic and pro-inflammatory. The depletion of CD169+ cardiac-resident macrophages by CD169-DTR and DT administration successfully depletes the Ccr2− cardiac-resident macrophage population [36]. The depletion of Ccr2− cardiac-resident macrophages before cardiac ischemia or cardiac stress by isoproterenol leads to increased mortality, fibrosis, and hypertrophy [10,51]. There is an increased inflammasome activation in the myocardium and IL1β expression, which exacerbates the initial injury [51]. On the contrary, Ccr2+ cardiac-resident macrophages promote an adverse outcome after cardiac injury. This population mediates neutrophil and Ccr2+ Cx3cr1^lo^ Ly6c^hi^ monocyte infiltration into the heart after cardiac injury, as demonstrated by experiments where Ccr2+ cardiac-resident macrophages were depleted using the mouse line Ccr2−DTR and DT administration, which resulted in reduced neutrophil and Ccr2+ Ly6c^hi^ monocyte infiltration into the heart after a subsequent injury [36]. This reduced infiltration of inflammatory leukocytes resulted in the reduced expression of inflammatory markers IL1β, IL6, and Tnfα and chemokines Ccl2 and Ccl7 in the heart [40]. Pharmacological blockade and genetic depletion of Ccr2 results in similar outcomes, which suggest that Ccr2+ cardiac-resident macrophages induce inflammatory monocyte infiltration by Ccl2 chemokine secretion [36]. Following the acute phase of monocyte infiltration and differentiation into inflammatory macrophages, a reparative phase takes place which is characterized by the infiltration and expansion of macrophages with reparative and fibrotic phenotype [69,75,76]. Meanwhile, macrophages with inflammatory phenotypes largely decrease primarily by local apoptosis, but a subset leaves the heart and is traced to secondary hematopoietic niches [26]. Most macrophages with reparative and fibrotic phenotypes seem to be derived from the initial Ccr2+ Cx3cr1^lo^ Ly6c^hi^ monocyte infiltration [76], as opposed to developing from the “patrolling” Ccr2− Cx3cr1^hi^ Ly6c^lo^ monocyte infiltration, which is described as anti-inflammatory and as mediating homeostatic functions, reviewed in [87,88]. This was demonstrated by experimental mice that had normal or elevated levels of Ccr2+ Cx3cr1^lo^ Ly6c^hi^ monocytes but lacked patrolling monocytes. Cardiac ischemia in these mice resulted in increased inflammatory macrophage abundance in the heart early after injury, but also increased reparative and pro-fibrotic macrophage abundance in the heart during the reparative phase [76]. It is unknown if cardiac-resident macrophages adopt different activation states and phenotypes during the different phases after myocardial infarction and ischemic heart failure development. However, scenarios where cardiac-resident macrophages dominate the immunological response after cardiac injury are characterized by minimal or no heart failure development, such as in the neonatal mouse or the zebrafish. In these scenarios cardiac-resident macrophages demonstrate a predominantly angiogenic, anti-inflammatory and non-fibrotic response [40,89,90,91], as discussed later. Although preliminary research which examines cardiac-resident macrophage response in myocardial infarction and ischemic heart failure is very promising, additional and more mechanistic experiments are needed to understand this response in detail.

### 5.2. Angiotensin Infusion and Pressure Overload

Angiotensin II infusion leads to the proliferation of Ccr2− and Ccr2+ cardiac-resident macrophages. Additionally, angiotensin II administration leads to an increased production of interleukin 1 beta (IL-1β) by Ccr2+ cardiac-resident macrophages and Ccr2+ Cx3cr1^lo^ Ly6c^hi^ monocytes [17]. Angiotensin II infusion promotes monocyte mobilization from splenic and bone marrow pools to the circulation [69,83,84,92], which suggest that chronic elevated levels of angiotensin II can lead to increased mobilization of monocytes from the bone marrow and other extramedullary tissues into the circulation, and potentially increase monocyte seeding in multiple organs, including the heart. Multiple pathological scenarios can potentially lead to increased levels of angiotensin II, such as ischemic heart failure or hypertension, and subsequently increase monocyte infiltration into the myocardium. An augmented influx of monocytes into the heart can permanently change the composition of cardiac-resident macrophages from a predominantly embryonic-derived scenario to a predominantly monocyte-derived scenario. Thus, this change in the composition of cardiac-resident macrophages in ischemic heart failure, hypertension, or cardiomyopathy is a mechanism worth exploring, as it could explain in part the pathophysiological progression and complications of these conditions.

Aortic or pulmonary artery banding results in an acute massive increase in afterload and pressure overload. As a result, there is a concentric hypertrophic response of the myocardium in order overcome such afterload [93]. Cardiac resident macrophages with low expression of Ly6c (Ly6c^lo^) were found to be critical mediators of this hypertrophic response by the CMs [93]. Amphiregulin (Areg) was identified by the authors as a secreted protein from cardiac-resident macrophages that promoted this hypertrophic response. Interestingly, colony stimulating factor 2 (Csf2) secreted by endothelial cells in the outer medulla of kidney after the acute cardiac pressure increase was acting on cardiac-resident macrophages and promoting the release of Areg by the latter [93]. The depletion of cardiac-resident macrophages or genetic depletion or Areg prevented the hypertrophic response, however, this resulted in a great increase in mortality [93]. Subsequent research by the same group demonstrated that Areg promotes phosphorylation of Cx43 which results in the proper formation of gap junctions among CMs, and enhances cardiac conduction [66], thus, the depletion of cardiac-resident macrophages or Areg not only blunts the hypertrophic response after pulmonary artery banding but also leads to fatal cardiac arrhythmias development [66,93].

### 5.3. Graft Rejection Following Heart Transplant

Cardiac-resident macrophages have also been identified to play a role in donor allograft injury during cardiac transplantation [94]. In this scenario the donor organ is unavoidably subjected to a temporary ischemic time that might result in ischemic damage, in addition to the acute and chronic immunological rejection of the allograft [95]. Cardiac-resident macrophages have a beneficial role in allogenic cardiac transplantation. Timd4+ cardiac-resident macrophages were identified as poor activators of effector T-cells, and instead promoted the activation of immunomodulatory T-regs [96] after allogenic heart transplants in mice. Interestingly, Timd4 expression itself promotes apoptosis of cardiac-resident macrophages, impairing their immunomodulatory effect and shortening graft rejection time [96]. Regulation of the immunological response during cardiac transplantation and other forms of cardiac injury, such as ischemic heart failure or hypertension, is an important area of research since the T-cell effector response contributes to cardiac remodeling in stress-overloaded and ischemic models of cardiac injury [97,98]. Cardiac resident macrophages could mediate an immunoregulatory effect in these scenarios as well, however, further research is needed to address this idea.

### 5.4. Chemotherapeutic Cardiotoxicity

Cardiac toxicity induced by anti-neoplastic chemotherapeutic compounds, specifically the anthracycline agent doxorubicin, has been broadly studied and described [43,99,100]. Doxorubicin therapy causes chemotherapy-induced cardiomyopathy (CICM) in as high as 30% of cancer survivors. Doxorubicin results in direct inhibition of DNA topoisomerase 2β, unrepaired DNA breaks, increased reactive oxygen species, and altered mitochondrial biogenesis in cardiomyocytes. Subsequently, the myocardium undergoes myofibrillar loss and vacuolization which ultimately leads to dilated cardiomyopathy [99]. Recent studies show that macrophage response in CICM is similar to the response after ischemic cardiomyopathy, with an influx of circulating monocytes into the myocardium and differentiation into inflammatory macrophages [42]. Using cell tracing, parabiosis, and stem-cell transplant experiments, it was demonstrated that cardiac-resident macrophages are initially reduced in proportion during CICM but they subsequently recover. During homeostasis, cardiac-resident macrophages that are maintained by proliferation in situ represent 80% of the total cardiac macrophage pool, decrease to 20% after 1 week of doxorubicin treatment, but recover to 50% of the cardiac macrophage pool 4 weeks after doxorubicin discontinuation [42]. Global macrophage depletion improves outcomes in a CICM mouse model, suggesting that macrophages generally contribute to damage progression in CICM [42]. Nonetheless, it seems that cardiac-resident macrophages have a beneficial effect in CICM. The Scaf1-Myc signaling pathway promotes macrophage proliferation in situ [42]. The blockade of this pathway primarily affects cardiac-resident macrophage response since monocyte-derived macrophage response depends on monocyte infiltration rather than proliferation in situ. The disruption of macrophage proliferation by a Scaf1-Myc blockade enhances CICM, results in an increased monocyte infiltration, and increases the expression of inflammatory markers IL1β, Tnfα, and IL6 in the heart. This suggests that cardiac-resident macrophage response is anti-inflammatory and counterbalances monocyte-derived macrophage response in CICM. However, the mechanisms behind the interaction between these two macrophage populations remain unclear.

## 6. Cardiac Macrophages in Cardiac Regeneration

It is well known that macrophages are involved in wound healing and inflammation resolution in multiple organs such as the gut, lungs, and skin, among others. There is variability regarding the contribution to the overall injury resolution process by tissue-resident macrophages among different organs, in addition to differences in the activation state and transcriptomic profile of these macrophages. Nonetheless, tissue-resident macrophages seem to contribute to injury resolution and the re-establishing of tissue homeostasis [101,102,103,104,105]. Less is known about cardiac-resident macrophages compared to infiltrating monocytes in the heart as a response to injury, but emerging literature has unveiled cardiac-resident macrophages as key mediators of cardiac injury resolution [10,36]. The use of pro-regenerative models such as the neonatal mouse or zebrafish enables identification of mechanisms that regulate cardiac regeneration that are absent or diminished in non-regenerative models such as adult mice and humans. These models have led to the identification of cardiac-resident macrophages as key mediators of cardiac regeneration [40,89,90,91].

Cardiac regeneration in zebrafish was first demonstrated in the early 2000s [106]. After the resection of the ventricular apex (roughly 20% of the ventricular mass) zebrafish are capable of complete cardiac regrowth without the development of fibrosis or heart failure. Neonatal mice also have a transient cardiac regenerative capacity within the first week of life, which is mediated by the proliferation of pre-existing CMs [107,108]. Complete neonatal heart regeneration has been demonstrated in the mouse after several types of injury including apical resection, ischemic cardiac injury, and cryoinjury [109,110,111,112]. While CM proliferation is critical for a successful neonatal cardiac regeneration, other cell types certainly contribute to this regenerative phenotype. The contribution of macrophages in cardiac regeneration was tested first by Aurora et al. using the neonatal mouse ischemic cardiac injury model [89]. The depletion of phagocytic cells, including macrophages by clodronate liposomes administration, impaired cardiac regeneration and decreased the density of microcapillaries in the infarcted and peri-infarcted myocardium [89]. Macrophage response after cardiac injury in neonatal mice is mediated by Ccr2− cardiac-resident macrophages, which proliferate in situ, and there is minimal contribution by infiltrating monocytes [40]. These cardiac-resident macrophages in neonatal mice have reduced expression of inflammatory genes IL1β and Tnfα compared to monocyte-derived macrophages isolated from adult mice. These studies also corroborated an increased angiogenic potential in cardiac-resident macrophages [40]. Inhibition of monocyte infiltration to the heart after cardiac injury by a Ccl2-Ccr2 signaling blockade leads to the increased proliferation of Ccr2− cardiac-resident macrophages, improved survival, functional recovery, a reduced ischemic area, and cardiac hypertrophy in adult mice [17,36,40].

A recent study that compared the transcriptomic profile from macrophages before and after cardiac injury, in regenerative P1 neonatal mice and non-regenerative P7 in adult mice, found that macrophages from adult mice demonstrated the differential expression of 1500 genes compared to macrophages from uninjured adult mice. Meanwhile, macrophages from neonatal mice differentially expressed only 398 genes compared to macrophages isolated from uninjured neonatal mice, while macrophages from P7 mice differentially expressed 470 genes compared to macrophages from uninjured P7 mice [90]. These data suggest that the macrophage response in adult mice is more complex. This large difference in transcriptional profiles may be explained at least in part by the massive addition of monocyte-derived macrophages to the adult heart after injury, compared to differences within cardiac-resident macrophages before and after injury in the P1 and P7 mice. The comparison of transcriptional patterns between macrophages from injured P1 and P7 mice demonstrated distinct transcriptional programs, which may underlie differences in the cardiac-resident macrophage responses; however, this remains to be further explored. Differentially expressed genes in macrophages isolated from adult hearts compared to macrophages isolated from neonatal hearts suggest not only a less inflammatory and pro-angiogenic response in macrophages from neonatal mice, but also a less fibrotic response [90]. Additional experiments demonstrated that macrophages present in injured hearts from adult mice could synthesize and deposit collagen, while macrophages from neonatal mice could not [90]. Interestingly, the transferring of monocytes from adult mice spleens to neonatal mice hearts at the time of injury resulted in a fibrotic response in neonatal mice, which demonstrated that monocyte-derived macrophages were highly fibrotic regardless of the microenvironment [90]. Cardiac-resident macrophages seemed to maintain this anti-fibrotic phenotype as demonstrated by depletion experiments [10,36], and experiments that led to an increased monocyte infiltration to the heart after injury, such as annexin 1 (AnxA1) depletion, a glucocorticoid-regulated protein that mediated immunoregulatory effects [113] and led to an impaired angiogenic macrophage response [81].

Studies in zebrafish also demonstrated a role for tissue-resident macrophages in promoting heart regeneration [91,114,115,116]. The ablation of the colony stimulating factor 1 receptor (Csf1r) homologue in zebrafish greatly depleted tissue-resident macrophages, but not macrophages present in the caudal hematopoietic tissue [116]. This depletion model in zebrafish was similar to Csf1r gene deletion in rodents, which depleted tissue-resident macrophages derived from a yolk-sac, but not monocyte-derived macrophages [5,7,28,29,30,31,32]. The depletion of tissue-resident macrophages in zebrafish led to impaired tissue regeneration by failed modulation of inflammation, overactivation of reactive oxygen species, and the decreased abundance of profibrotic markers such a Tgfβ [116]. In contrast, depletion of caudal hematopoietic macrophages, which were equivalent to monocyte-derived macrophages in mammals, resulted in normal heart regeneration despite the overall decrease in macrophage infiltration to the site of injury [116].

Using models capable of cardiac regeneration, like neonatal mice and zebrafish, demonstrated important differences in macrophage responses after cardiac injury between regenerative and non-regenerative models. Regenerative models had a predominantly cardiac-resident macrophage response, which was less inflammatory, more angiogenic and less fibrotic than monocyte-derived macrophages, which dominate the macrophage response in adult mice and humans. There is little known about the interactions between cardiac-resident and monocyte-derived macrophages in general, and further research is needed to understand the dynamics that govern these interactions after cardiac injury, which makes this a promising area of research.

## 7. Future Perspectives

Cardiac-resident macrophages are becoming an exciting area of research, since recent literature demonstrates the beneficial functions that these cells have during cardiac development, cardiac homeostatic function and in recovery following cardiac ischemia and other forms of cardiac injury. Future research should focus on the molecular, cellular, histological, and systemic mechanisms by which these cells mediate homeostatic functions, such as cardiac extracellular surveillance and exophere removal, or cardiac conduction enhancement through the phosphorylation of Cx43 and correct gap-junction conformation. An improved understanding of mechanisms, that modulate the function of cardiac-resident macrophages, will greatly impact fields such as ischemic heart failure development and progression, cardiac arrhythmias, heart transplantation tolerance, and cardiac chemotoxicity pathophysiology, among others. Identification of the molecules and mediators of the relationship between cardiac-resident macrophages, and other cell types in the heart in these pathological scenarios, could lead to the development of pharmacological inhibitors or enhancers that target the pathophysiology of these processes from a novel perspective.

## 8. Conclusions

Cardiac-resident macrophages constitute a heterogenous group of subpopulations with overlapping and distinct functions which are important in cardiac development, homeostasis, and recovery after cardiac injury. Embryonic-derived populations are important for coronary and lymphangiogenic development and maturation, immunological surveillance of the heart and maintenance of the extracellular compartment. Additionally, embryonic derived macrophages promote angiogenesis and cardiac regeneration after injury. Conversely, populations that are monocyte-derived are important for antigen presentation and the activation of T-cells and are key initiators of the inflammatory response after cardiac injury. Understanding the mechanisms that control cardiac-resident macrophage function is important in order to understand cardiac development and homeostatic functions. Additonally, from the pathophysiological perspective of multiple cardiac diseases such as arrhythmias and heart failure, a better understanding of these cells creates a promising field of research and a possible target to treat such conditions.

## Figures and Tables

**Table 1:** Cardiac-resident macrophage clusters by surface protein, origin, maintenance, and functions.

Cell Type	Surface Markers	Origin	Maintenance	Functions
Cardiac resident macrophages	Ccr2− Timd4+ MHCII^lo^	Embryonic	Proliferation in situ >90%	Homeostatic Phagocytic Coronary development Lymph-angiogenesis
Cardiac resident macrophages	Ccr2− Timd4− MHCII^hi^	Embryonic	Proliferation in situ 75%	Homeostatic Antigen presentation to T-cells
Cardiac resident macrophages	Ccr2+ Timd4− MHCII^hi^	Monocytes	Proliferation in situ 15–20%	Monocyte and neutrophil chemo-taxis post-injury
Monocytes	Ccr2+ Timd4− MHCII^lo^	Monocytes	Monocyte infiltration >99%	Ccr2+ macrophage cluster replenishment

**Table 2:** Cardiac-resident macrophage clusters in homeostasis by transcriptomic profile.

Cell Type	Cluster	Signature Genes	Origin	Functions
Cardiac resident macrophages	scRNAseq 1	Timd4 Folr2 Lyve1 CD163 Igf1	Embryonic	Homeostatic Angiogenesis Endocytosis Lysosomal
Cardiac resident macrophages	scRNAseq 2	MHC II F4/80 Cx3xr1 CD14	Embryonic	Translational Ribosomal Antigen presentation
Cardiac resident macrophages	scRNAseq 3	Ccr2 CD64	Monocytes	Classical inflammatory pathways
Cardiac resident macrophages	scRNAseq 4	Ifit1 Ifit3 Irf7	Monocytes	Classical inflammatory pathways Interferon response
